# Evaluation of the Phytochemical Screening of Methanolic Seed Extracts of Tribulus terrestris: An In Vitro Application of Anti-cancer, Anti-oxidant, and Anti-microbial Activities

**DOI:** 10.7759/cureus.66674

**Published:** 2024-08-12

**Authors:** Mohammed Yaseen Malik, Arockia Alex, Azhagu Madhavan Sivalingam, Brahma Neha, Sugumar Vimal

**Affiliations:** 1 Biochemistry, Saveetha Medical College and Hospital, Saveetha Institute of Medical and Technical Sciences (SIMATS), Chennai, IND; 2 Community Medicine/Diabetes and Endocrinology, Pharmacology, Nanotechnology, Saveetha Medical College and Hospital, Saveetha Institute of Medical and Technical Sciences (SIMATS), Chennai, IND

**Keywords:** anti-microbial, anti-inflammatory, anti-cancer, phytochemicals, tribulus terrestris

## Abstract

Background: *Tribulus terrestris*, a plant known for its pharmacological properties, was investigated in this study for its potential anticancer effects against oral cancer cells. The study aimed to explore the phytochemical composition of *T. terrestris *seed extract and evaluate its cytotoxic, pro-apoptotic, antioxidant, anti-inflammatory, and antimicrobial activities.

Materials and methods: Methanolic seed extracts of *T. terrestris *were obtained and subjected to phytochemical analysis to identify bioactive compounds. The cytotoxic effect of the extract on oral cancer cells was evaluated using the MTT (3-(4, 5-dimethylthiazolyl-2)-2, 5-diphenyltetrazolium bromide) assay, while pro-apoptotic effects were assessed through dual fluorescent staining. Antioxidant activity was measured using hydrogen peroxide and erythrocyte aggregation assays, while anti-inflammatory activity was evaluated through inhibition of albumin denaturation.

Results: Phytochemical analysis revealed the presence of alkaloids, tannins, saponins, flavonoids, and phenols in *T. terrestris* seed extract. The extract demonstrated concentration-dependent cytotoxicity against oral cancer cells, with 100 μg/mL showing significant growth inhibition. Pro-apoptotic effects were observed, with characteristic morphological changes in cancer cells treated with the extract. Antioxidant activity was demonstrated by the extract, with methanol fraction of a flower (MFF) exhibiting the highest capacity, followed by total trichome fraction (TTF), and a positive correlation between phenolic content and free radical scavenging effectiveness was noted. Antimicrobial activity against various pathogens, including bacteria and fungi, was also observed, with higher concentrations showing increased efficacy.

Conclusion: The study concludes that methanolic extracts of* T. terrestris *possess significant anticancer, antioxidant, anti-inflammatory, and antimicrobial activities. These findings highlight the potential of *T. terrestris *as a candidate for further research and clinical applications, either alone or in combination with other agents, for the treatment of oral cancer and associated conditions.

## Introduction

*Tribulus terrestris*, commonly known as puncture vine or devil's weed, is a plant deeply rooted in traditional medicine, renowned for its potential to enhance athletic performance, improve sexual function, and address various health concerns [1]. This versatile plant, often marketed as a natural testosterone booster, offers a spectrum of health benefits, including anti-inflammatory and antioxidant properties that can safeguard the body against the damaging effects of free radicals [2]. However, its effectiveness as a testosterone booster remains subject to mixed research findings [3]. *T. terrestris's* diuretic properties can potentially increase urine output and reduce water retention, making it of interest to those with specific health needs [4]. Moreover, its historical reputation as an aphrodisiac is believed to enhance libido and sexual function in both men and women [5]. Despite being generally considered safe, it is advisable to consult a healthcare provider before using *T. terrestris*, especially when taking certain medications, as it can cause side effects such as stomach upset, headaches, and sleep disturbances [6]. Beyond its traditional uses, modern research has unveiled additional facets of its health potential, including antibacterial effects against oral pathogens and protection against heavy metal toxicity, oxidative stress, cytotoxicity, and helminthic infections [7]. Its fruit, employed in ayurveda to address hypertension, eye problems, oedema, sexual dysfunction, and urinary system disorders, contains various bioactive compounds such as alkaloids, flavonoids, tannins, and glycosides, which influence physiological and pathological conditions [8]. Furthermore, *T. terrestris *serves as a natural source of antioxidants, abundant in its fruits, leaves, and flowers, which are renowned for their ability to scavenge free radicals, considered less hazardous than synthetic counterparts [9]. Medicinally, it has been used in combination with other substances to reduce blood pressure and cholesterol levels [10]. Beyond these applications, this plant has emerged as a potential anticancer agent, exhibiting efficacy against various cancer types, including breast and prostate cancer, with specific compounds such as nuatigenin contributing to its anticancer properties [11,12]. Its hydroalcoholic extract from fruits and seed extract demonstrates promise in combating cancer cell lines [13,14]. *T. terrestris, *belonging to the* Zygophyllaceae *family, thrives in diverse regions across Europe, Asia, Africa, and Australia [15], adapting to harsh conditions, which has contributed to its resilience and popularity in the fitness and wellness industry due to its potential health benefits, particularly its association with boosting testosterone levels and enhancing athletic performance [16]. Its availability in various forms, from capsules to powders and tinctures, enables individuals to harness its health-promoting potential, providing a multifaceted approach to well-being that bridges traditional remedies with contemporary medicinal research, offering novel opportunities for synergistic therapeutic breakthroughs. This study focuses on the aim of the *in vitro *anticancer and antimicrobial pharmacological application of biological activity of antipyretic properties.

## Materials and methods

Collection of plant

*T. terrestris *plant seeds were gathered from Chennai, Tamil Nadu, India. The collected plant seed material was shade-dried and made into powder. Then, 500 g of plant powder was kept in a Soxhlet extractor, and methanol (1,250 mL) was added and extracted for two hours and then filtered with Whatman filter paper (No. 41). It is concentrated by rotating the evaporator at 60°C. The concentrated crude plant methanol extract is stored at -20°C for future analysis.

Qualitative phytochemical (metabolites) analysis of *T. terrestris*

Secondary metabolite analysis of *T. terrestris's* methanol seed extract was focused on confirmatory analysis, either the presence or absence of bioactive secondary metabolites [17]. It focused on tannins, saponins, flavonoids, alkaloids, and polyphenols, and the standard biochemical protocols were followed in accordance with established previously procedures [18].

Cell culture reagents and cell line maintenance

Dulbecco's modified eagle medium (DMEM), fetal bovine serum (FBS), trypsin-ethylenediaminetetraacetic acid (EDTA), and phosphate-buffered saline (PBS) were procured from Gibco in Canada. Acridine orange (AO), ethidium bromide (EtBr), dimethyl sulfoxide (DMSO), MTT ([3-(4,5-dimethylthiazol-2-yl)-2,5-diphenyl tetrazolium bromide), and DAPI (4',6-diamidino-2-phenylindole) were obtained from Sigma Chemical Pvt. Ltd. in the USA. The remaining chemicals were purchased from SRL in India, and they were of extra-pure molecular grade. We procured the KB-1 oral cancer cell line from the National Centre for Cell Science (NCCS) in Pune. To culture the bacteria, T25 culture flasks were utilized, which contained a mixture of DMEM, along with small quantities of 10% FBS and 1% penicillin-streptomycin antibiotics. These cells were carefully maintained at a temperature of 37°C in a humid atmosphere with a 5% CO_2 _concentration. Upon reaching confluency, the cells were trypsinized and subsequently passaged [19].

In vitro cell viability assay by MTT

The MTT assay was conducted on the KB-1 cell line to assess cell viability in samples treated with *T. terrestris.* The underlying principle of this assay relies on the metabolic activity of cells, which reduces a soluble yellow tetrazolium salt into insoluble purple formazan crystals. Initially, 96-well plates were prepared and loaded with approximately 5x10^3 ^cells/well, followed by a 24-hour incubation period. Subsequently, the cells were washed with 100 μL of serum-free medium and placed in a 37°C environment. The details of the protocol of the cell viability assay are described by Vimal et al. [20].

Determination of Mode of Cell Death by AO/EtBr Dual Staining

The cells were subjected to a 24-hour treatment with *T. terrestris*, after which they were harvested and washed with PBS. The cell pellets were subsequently resuspended in approximately 5 µL of AO (1 mg/mL) and 5 µL of EtBr (1 mg/mL). The prepared samples were then examined under a fluorescence microscope [21].

In vitro anti-oxidant activity: H_2_O_2_ assay

The H_2_O_2_ assay for antioxidant activity involved the preparation of fresh solutions. A 1.0 mL reaction mixture was prepared, consisting of approximately 100 µL of 28 mM 2-deoxy-2-ribose (dissolved in phosphate buffer, pH 7.4), 500 µL of a solution with varying concentrations (10 µL, 20 µL, 30 µL, 40 µL, and 50 µL), 200 µL of 200 µM FeCl_3_, 1.04 mM EDTA (in a 1:1 v/v ratio), 100 µL of H_2_O_2_ (1.0 mM), and 100 µL of ascorbic acid (1.0 mM). After incubating for one hour at 37°C, the extent of deoxyribose degradation was measured at approximately 532 nm, comparing the results to a blank solution [22].

In vitro anti-inflammatory activity: egg albumin denaturation assay

The assessment of Oolong tea gel's anti-inflammatory activity followed a protocol. In this method, 0.05 mL of gel at various concentrations (10 µL, 20 µL, 30 µL, 40 µL, 50 µL) was mixed with 0.45 mL of bovine serum albumin (1% aqueous solution). The pH of the mixture was adjusted to approximately 6.3 using a small quantity of 1N HCl. Subsequently, these samples were left to incubate at room temperature for about 20 minutes, followed by a 30-minute heating at 55°C in a water bath. After cooling, the absorbance of the samples was measured spectrophotometrically at 660 nm. Diclofenac Sodium served as the standard reference, and DMSO was used as the control. The percentage of protein denaturation was calculated using the formula: % inhibition = (Absorbance of control - Absorbance of the sample) / Absorbance of control × 100 [23].

In vitro antimicrobial activity of methanolic extract of *T. terrestris* was done by the well diffusion method 

The procedure began by sterilizing Mueller Hinton agar medium at 121°C for 15 minutes at 15 lbs of pressure. Once sterilized, the medium was poured into sterile petri plates and allowed to solidify. Five sterile petri plates were prepared for the experiment. Pathogens, namely *Streptococcus mutans, Staphylococcus aureus, Enterococcus faecalis*, and *Candida albicans*, which had been cultured for 16 hours, were swabbed onto the surface of the medium using a sterile cotton swab. After a 10-minute incubation period, wells were created in the agar medium using a cork borer. The seed extracts were prepared in 20% DMSO at concentrations of 25 µg/mL, 50 µg/mL, and 100 µg/mL, with each concentration loaded into four different wells. Additionally, 20% DMSO was used as a control. The zones of inhibition were subsequently measured [24].

Statistical analysis

The analysis of variance was performed, followed by the use of GraphPad Prism (version 7.0; GraphPad Software, San Diego, CA), and standard deviation was used. The significance level was set at <0.05 for the analysis of cell growth inhibition.

## Results

Phytochemical analysis

The phytochemical analysis conducted in this study revealed the presence of significant bioactive compounds, such as alkaloids, tannins, saponins, flavonoids, and phenols. These secondary metabolites are widely recognized for their anti-cancer and anti-inflammatory properties (Table 1). The biochemical application of these metabolites in the plant resources of *T. terrestris* can play a significant role in combating human pathogenic microorganisms.

**Table 1:** Secondary metabolites plant seed extract Tribulus terrestris using methanol solution A single "+" indicates a detectable presence of the metabolite. A double "++" indicates a higher concentration or a stronger presence of the metabolite compared to a single "+".

S. No	Secondary metabolites	*Tribulus terrestris *methanol extract
1	Alkaloids	+
2	Tannins	++
3	Saponins	+
4	Flavonoids	++
5	Phenols	+

In vitro cytotoxicity by the MTT assay

**Figure 1:**
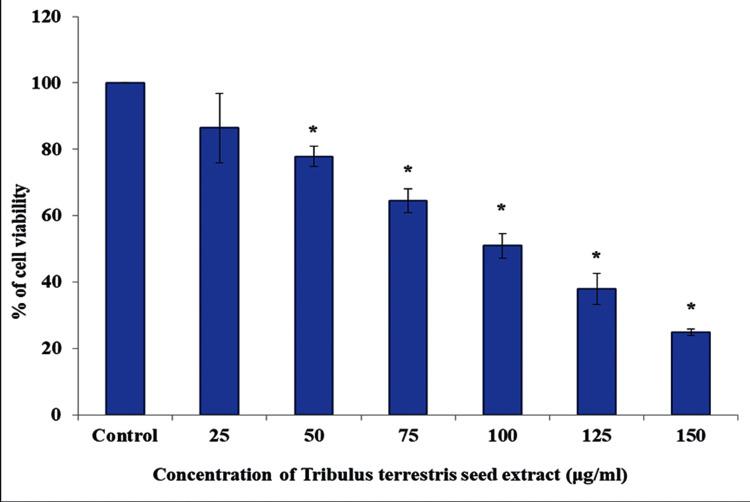
In vitro cytotoxicity by the MTT assay

The in vitro cytotoxic effect of *T. terrestris* seed extract on oral cancer cells was assessed by the MTT assay. For 24 hours, oral cancer cells were treated with different concentrations of *T. terrestris* seed extract (0, 25, 50, 75, 100, and 150 μg/mL). The percentage of cell viability decreased steadily as concentration increased. At 100 μg/mL concentration, we observed 50% growth inhibition (Figure 1). As a result, the IC 50 dose (100 μg/mL) was chosen for future investigations.

In vitro evaluation of the pro-apoptotic effect of *T. terrestris* seed extract in oral cancer cell line (dual staining)

**Figure 2:**
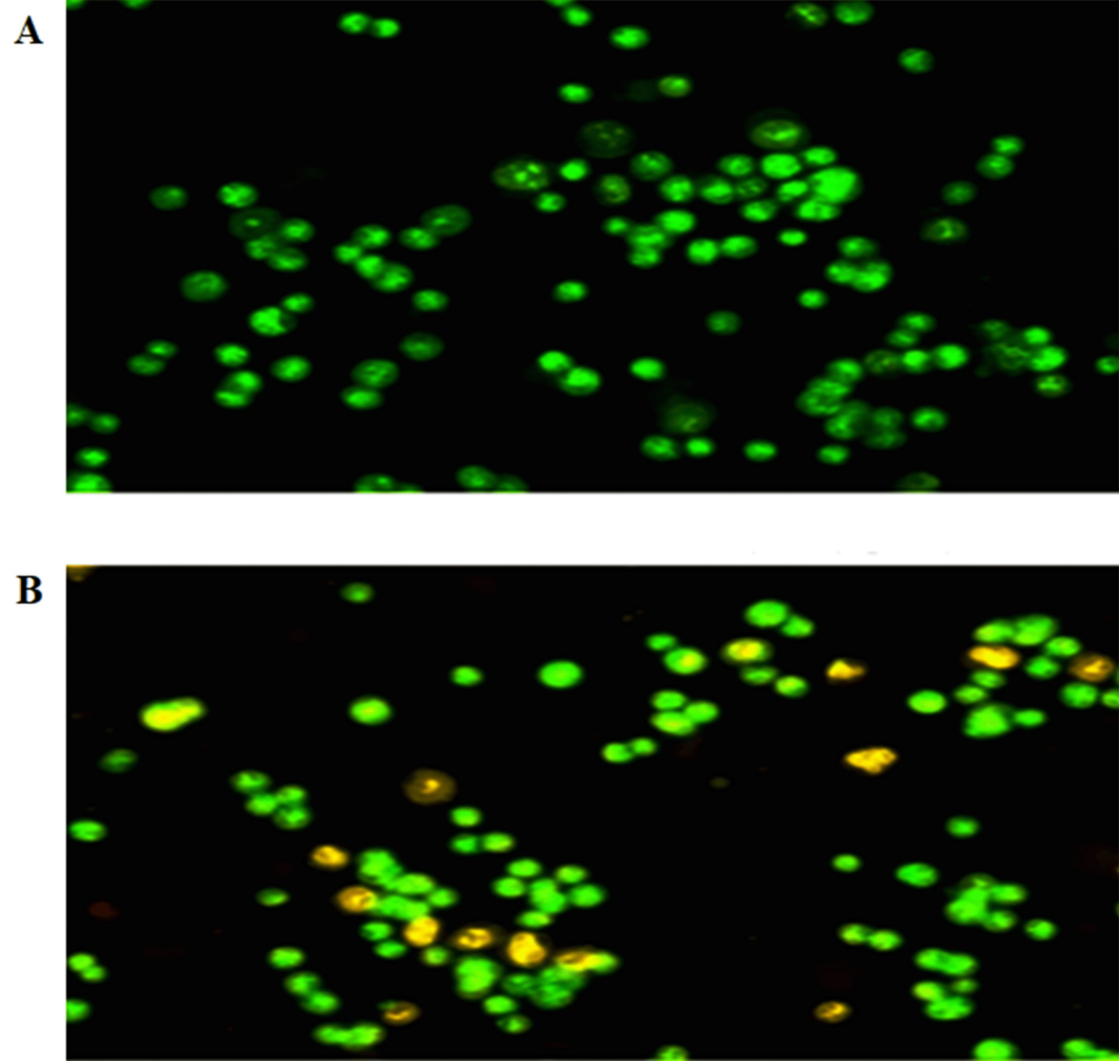
Pro-apoptotic effect of Tribulus terrestris seed extract in oral cancer cell line (dual staining) A. Control Cells, B. Cells were treated with methanolic extract of Tribulus terrestris (100 µg/mL) for 24 h along with the control group. Images were obtained using an inverted fluorescence microscope.

The induction of apoptosis in cells treated with *T. terrestris *seed extract (100 μg/mL) was examined using dual fluorescent staining solution (1 µL) containing 100 µg/mL AO and 100 µg/mL EB solution. The result revealed that cell shrinkage and cytoplasmic membrane blebbing were observed as morphologic alterations viewed under a fluorescent microscope (Figure 2).

In vitro anti-oxidant response by the H_2_O_2_ assay

**Figure 3:**
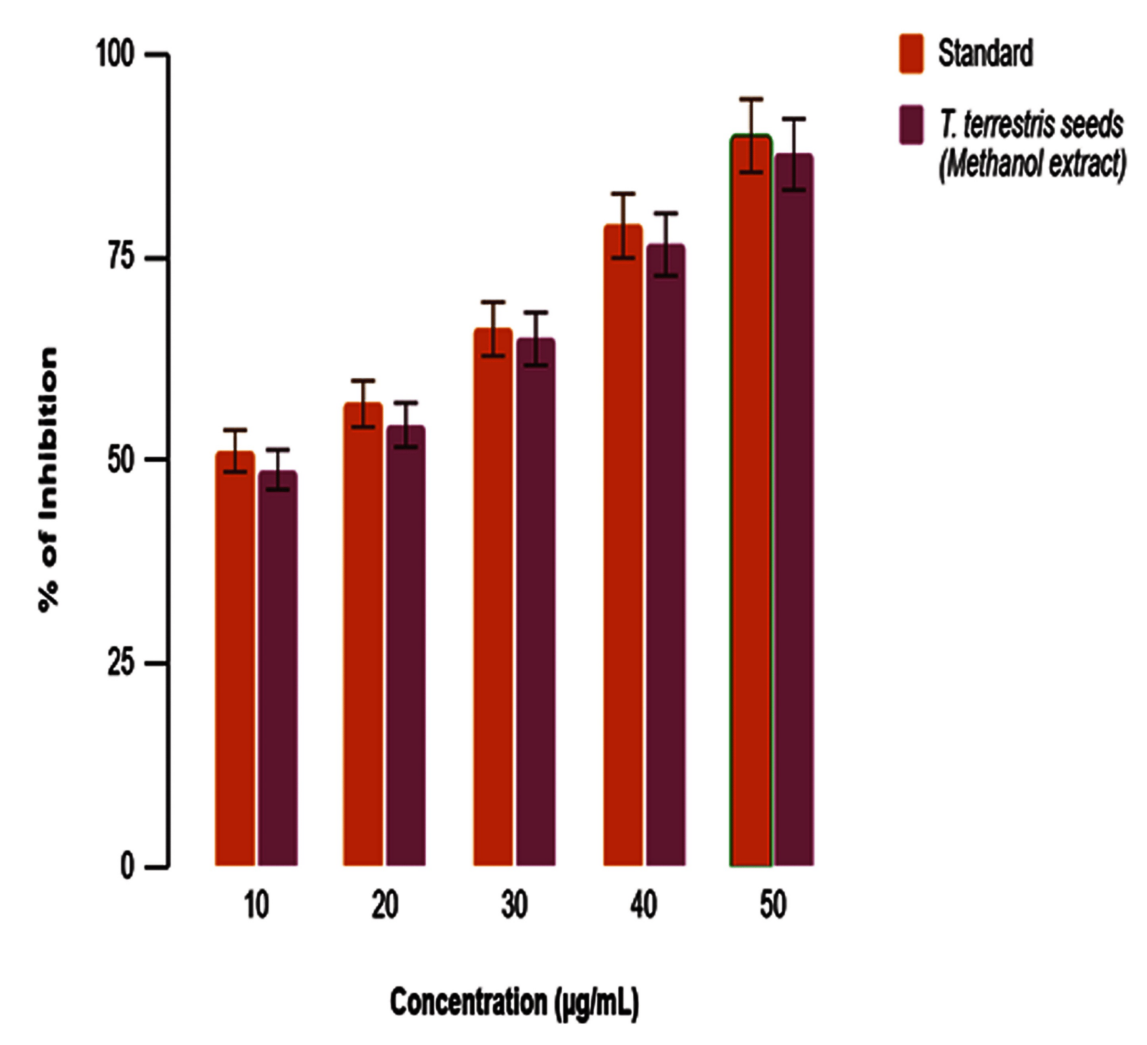
In vitro anti-oxidant activity of methanolic extracts of Tribulus terrestris by the H2O2 assay

The graph (Figure 3) presents a comparison of the inhibition percentage at different concentrations (10, 20, 30, 40, and 50 µg/mL) between a standard reference compound and the methanol extract of *T. terrestris* seeds. The y-axis depicts the degree of inhibition expressed as a percentage, while the x-axis displays the concentration measured in micrograms per milliliter (µg/mL). Notable findings include a rise in inhibition as the concentration increases for both the standard and the methanol extract. At a concentration of 10 µg/mL, the standard exhibits around 52% inhibition, while the methanol extract demonstrates approximately 50% inhibition. At a concentration of 20 µg/mL, both the standard and the extract exhibit a rise, with the standard showing an approximate 60% increase and the extract showing a somewhat smaller increase. At a concentration of 30 µg/mL, the standard achieves an approximate yield of 70%, which is comparable to the extract. At a concentration of 40 µg/mL, the standard exhibits an approximate efficacy of 80%, whereas the extract demonstrates a slightly lower efficacy. At a concentration of 50 µg/mL, both substances achieve approximately 90%. The error bars demonstrate more variability for the extract in comparison to the standard. Overall, the methanol extract demonstrates similar inhibitory effects to the standard, particularly at higher concentrations, indicating its efficacy in suppressing the measured activity. 

In vitro anti-inflammatory response by the egg albumin assay

**Figure 4:**
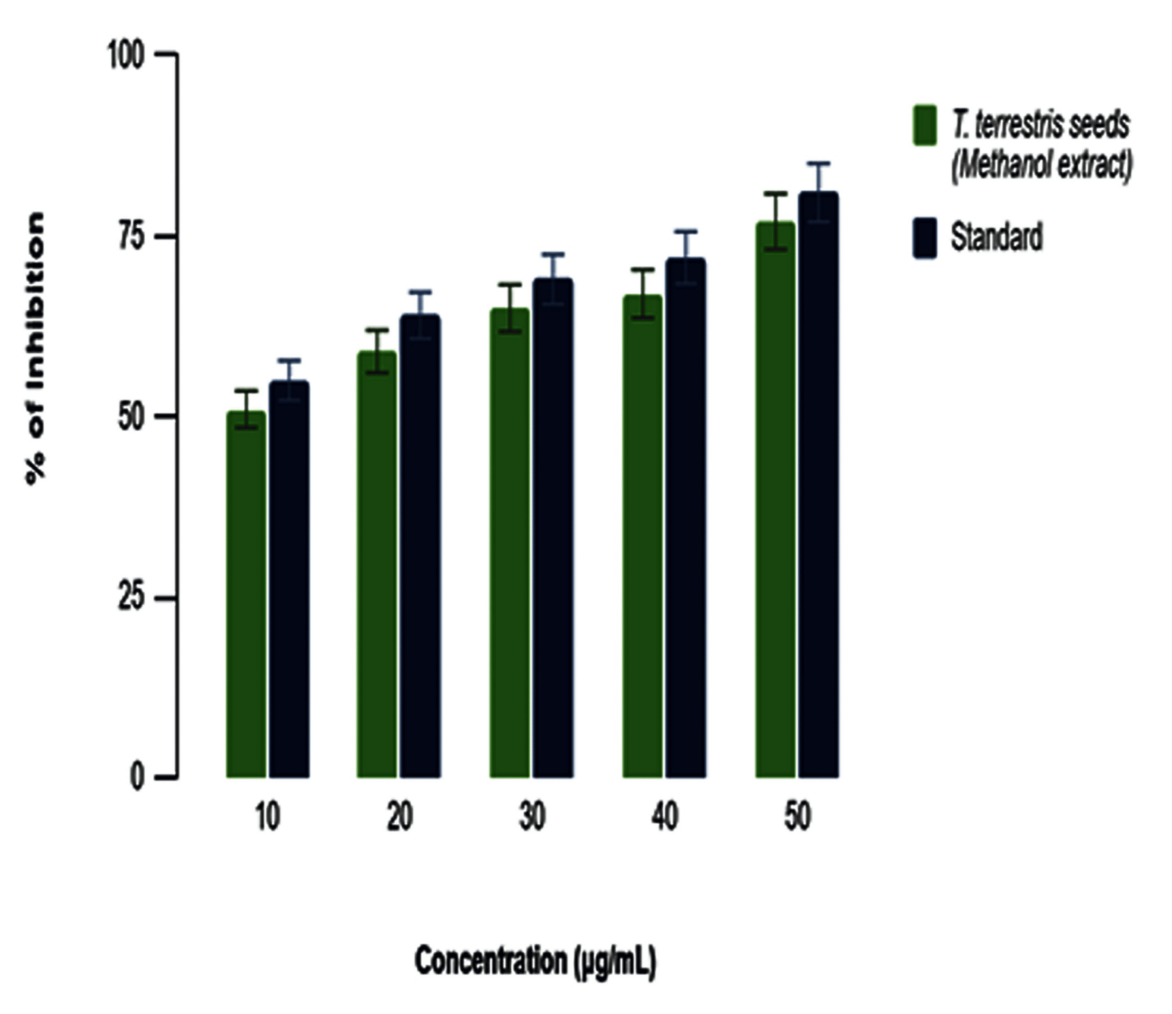
In vitro anti-inflammatory activity of methanolic extracts of Tribulus terrestris by the endotoxin activity (EA) assay

The graph (Figure 4) that shows the percentage of inhibition seen for different amounts of *T. terrestris *seeds methanol extract (10, 20, 30, 40, and 50 µg/mL) compared to a standard. The concentrations are graphed on the x-axis, while the percentage of inhibition is shown on the y-axis. At a concentration of 10 µg/mL, the methanol extract exhibits a little lower inhibition compared to the standard, with both showing approximately 50% inhibition. At a dose of 20 µg/mL, both the extract and the standard exhibit a steady inhibitory percentage, with a small rise that remains close to 50%. At a concentration of 30 µg/mL, the inhibition percentage of the methanol extract increases, approaching that of the standard. The effect lasts at concentrations of 40 and 50 µg/mL. At these levels, the methanol extract's inhibition percentage reaches its highest point and starts to be similar to the standard, getting close to 90%. The error bars represent the extent of variation in the results, which seem to be very small, indicating consistent outcomes across the replicates. In general, the methanol extract derived from *T. terrestris *seeds exhibits a rise in inhibition that is dependent on the concentration. At higher concentrations, it performs comparably to the standard.

Anti-microbial response

**Figure 5:**
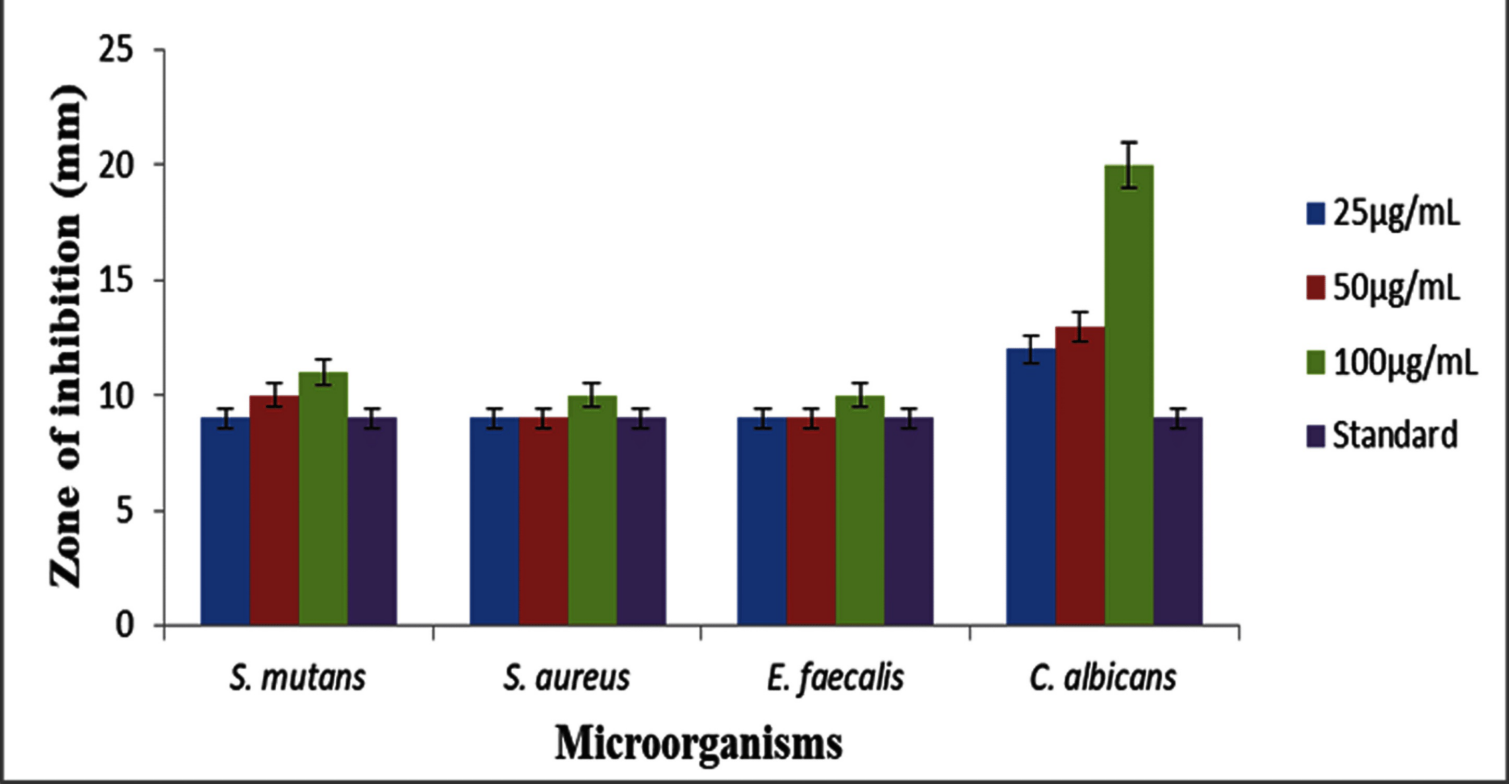
Zone of inhibition with methanolic seed extracts of Tribulus terrestris against oral pathogens

The graph (Figure 5) depicts the zones of inhibition for *S. mutans, S. aureus, E. faecalis,* and *C. albicans* at concentrations of 25 µg/mL, 50 µg/mL, and 100 µg/mL, in comparison to a standard. At all doses,* S. mutans* exhibited inhibitory zones ranging from roughly 11-13 mm, with the average being around 11 mm. The inhibition zones of *S. aureus *and *E. faecalis *were approximately 10-12 mm, which is comparable to the standard. *C. albicans* exhibited the most notable reaction, as the size of the inhibitory zones increased from 12 mm at a concentration of 25 µg/mL to 20 mm at a concentration of 100 µg/mL, whereas the standard measurement was around 11 mm. The solution with a concentration of 100 µg/mL showed significant efficacy against *C. albicans.*

## Discussion

*Tribulus terrestris,* *Silybum marianum*, and *Eichhornia crassipes *are plants known for their notable medical capabilities, primarily attributed to their bioactive components. *T. terrestris* is a plant that includes saponins, flavonoids, alkaloids, and glycosides. It is commonly used in traditional medicine to improve sexual desire, treat urinary tract problems, and promote cardiovascular well-being. It can be used to increase testosterone levels, control diabetes, and function as an anti-inflammatory medication. Silybum marianum, also referred to as milk thistle, contains a high concentration of silymarin, which is a mixture of flavonolignans, tannins, flavonoids, and polyphenols. This plant is renowned for its hepatoprotective characteristics and antioxidant advantages, with possible uses in suppressing leukotriene formation, regulating diabetes, treating chronic hepatitis, and demonstrating antipsoriatic activity. *E. crassipes,* commonly known as water hyacinth, flourishes in contaminatardsed bodies of water and can assimilate inorganic pollutants. The presence of polyphenolic chemicals, flavonoids, and tannins in this plant contributes to its potential therapeutic properties, comparable to other plants that have a high polyphenolic content. The connection between these plants is based on their common bioactive components, including flavonoids and polyphenols, which are responsible for their anti-inflammatory, antioxidant, and possibly anti-diabetic effects. Scientists can learn more about the healing properties, how they work, and what they bring to the field of natural medicine by looking at the benefits and bioactive parts of* T. terrestris* in comparison to *S. marianum *and *E. crassipes*. By employing a comparative approach, it is possible to uncover synergistic effects and thus augment the collective understanding of these invaluable therapeutic plants [25,26]. One of the most important non-communicable disorders is cancer. It is accountable for deaths everywhere [27]. According to the findings, cisplatin and methanolic extract caused significantly more DNA damage in treated cells than in untreated ones [13]. In the oral cancer cell line KB-1, *T. terrestri*s can cause apoptosis by lowering Bcl-2 protein levels, raising caspase-3 activity, and degrading DNA [28]. KB-1 cells are derived from a human nasopharyngeal carcinoma or an oral carcinoma treated with different concentrations of T. terrestris extracts for an in vitro anti-cancer study [29]. In this work, p53 levels significantly increased in KB-1 cells treated with* T. terrestris*, demonstrating that p53 is activated in early apoptosis [30]. As a result, it appears that KB-1 cells treated with *T. terrestris* extracts had a high induction of caspase-3 activity. It is seen that the influence of *T. terrestris* on the cancer cells was more significant than that on healthy fibroblasts, suggesting the selective application of this plant against cancer cells [31]. In another research, *T. terrestris* extract was seen to block the proliferation of the cancer cells and also to trigger the apoptosis of the human liver cancer cells through NF-κB signaling inhibition [32]. *T. terrestris* has shown a protective effect against UVB-induced carcinogenesis [5]. In many studies, steroidal saponins purified from *T. terrestris* was used against several cancer cell lines [33]. Alkaloids extracted from *T. terrestris*, viz., trans-N-feruloyl-3-hydroxytyramine and trans-N-feruloyl-3-ethoxytyramine, have been reported by articles to induce apoptosis in leukemic cancer cells [12]. *T. terrestris*-treated MCF-7 cells also showed a significant increase in p53, indicating their activation in early apoptosis. *T. terrestris* extract displayed its ability to stabilize human RBC membrane by inhibiting heat-induced hemolysis by showing consistency with its phytochemical and also antioxidant potential [34]. The protein denaturation bioassay was used for its in vitro assessment of the anti-inflammatory activity of *T. terristris* fruit extract. It is suggested that prostaglandin E is a mediator of the anti-inflammatory action of *T. terrestris* [35]. *T. terrestris* plant, according to researchers, can protect rat brains from inflammation, which was caused by formalin and carrageenan by the down-regulation of NF-κB [31]. Oxidative stress is a contributing factor to many chronic diseases, including cardiovascular diseases, neurodegenerative disorders, and cancer. Antioxidants in *T. terrestris*, such as flavonoids and polyphenols, promote overall cellular health by maintaining the integrity of cell membranes [36]. The combination of anti-inflammatory and antioxidant properties in *T. terrestris *may lead to improved cardiovascular health [37]. Antioxidant properties of *T. terrestris* plant seed extract support reproductive health by protecting sperm and eggs from oxidative damage [38]. *T. terrestris* is also seen to help regulate blood sugar levels, possibly due to its anti-inflammatory effects [39]. As a result, it can be utilized as an alternative form of treatment for individuals whose gentamicin dosage has been reduced or whose treatment duration has been shortened. *T. terrestris* can therefore be used as an alternative therapy for the treatment of urinary tract infections [40]. *C. albicans* continues to be the most prevalent infection-causing fungus; it is responsible for roughly 45% of clinical fungal infections [41]. Many compounds found in *T. terrestris* plant seed extracts, including saponins, may be responsible for the plant's antifungal properties [42]. Turkish *T. terrestris,* Iranian *T. terrestris, *and Iraqi *T. terrestris *plant seed extract have similar antibacterial properties. Various geographic sources of the plant used, the strains employed, and various assay techniques may all have an impact on the antibacterial activity of *T. terrestris. T. terrestris* is seen to protect against heavy metal toxicity, oxidative stress, cytotoxicity, and helminthic infections [43]. *T. terrestris* seed extract's anticancer activity as microbial plants or drugs should be explored in future research and its clinical applications.

Limitations and future studies

Despite its promising therapeutic potential, several limitations of *T. terrestris *warrant further investigation. The mixed research findings on its efficacy as a testosterone booster necessitate rigorous clinical trials. Its diuretic properties, while beneficial for reducing water retention, pose risks of dehydration and electrolyte imbalance. Side effects such as stomach upsets, headaches, and sleep disturbances highlight the need for medical consultation before use. Extracts' anticancer activity necessitates extensive validation through in vivo studies and clinical trials. The variability in bioactive compound concentrations necessitates standardized preparation protocols. The reliance on in vitro assays underscores the need for comprehensive in vivo studies to understand its pharmacokinetics, bioavailability, and long-term safety. Exploring synergistic effects with other medicinal plants, such as *S. marianum* and *E. crassipes,* could unveil new therapeutic avenues. Future studies should address these limitations through robust clinical trials, standardized extraction methods, and a deeper exploration of molecular mechanisms, ensuring the safe and effective use of *T. terrestris* in diverse medical applications.

## Conclusions

This study investigated the pharmacological capabilities of *T. terrestris,* specifically examining its potential as an anticancer and antibacterial agent in laboratory settings. It was found that the methanolic extract of *T. terrestris *seeds had bioactive substances such as alkaloids, tannins, saponins, flavonoids, and phenols that are known to fight cancer and inflammation. The extract demonstrated a cytotoxic impact on Kb-1 oral cancer cells that was dependent on the dosage. It achieved a 50% suppression of cell growth at a concentration of 100 µg/mL, and it suppressed cell growth by 50% and induced apoptosis. The H_2_O_2_ assay assessed the antioxidant activity, which exhibited 90% inhibition at a concentration of 50 µg/mL. The extract also exhibited potent anti-inflammatory properties and notable antibacterial activity, namely against *C. albicans*. The results emphasize *T. terrestris'* therapeutic potential, indicating the need for further research on its bioactive components for medical purposes.

## References

[1] Azam F, Munier S, Batool M, Ahmad B, Abbas G (2019). A review on advancements in ethnomedicine and phytochemistry of Tribulus terrestris-a plant with multiple health benefits. Int J Biosci.

[2] Chokkalingam P, Dhandapani H, Sekar K, Durairaj P, Hari R (2020). Enzymatic and nonenzymatic antioxidant activity of the saponin rich butanol extract of Tribulus terrestris fruits against tetrachlorodibenzo-p-dioxin induced oxidative stress in male Wistar rats. Biomedicine.

[3] Gunnels TA, Bloomer RJ (2014). Increasing circulating testosterone: impact of herbal dietary supplements. J Plant Biochem Physiol.

[4] Sudheendran A, Shajahan MA, Premlal S (2021). A comparative diuretic evaluation of fruit and root of Gokshura (Tribulus terrestris Linn.) in albino rats. Ayu.

[5] Zhu W, Du Y, Meng H, Dong Y, Li L (2017). A review of traditional pharmacological uses, phytochemistry, and pharmacological activities of Tribulus terrestris. Chem Cent J.

[6] Kamenov Z, Fileva S, Kalinov K, Jannini EA (2017). Evaluation of the efficacy and safety of Tribulus terrestris in male sexual dysfunction-a prospective, randomized, double-blind, placebo-controlled clinical trial. Maturitas.

[7] Chhatre S, Nesari T, Somani G, Kanchan D, Sathaye S (2014). Phytopharmacological overview of Tribulus terrestris. Pharmacogn Rev.

[8] Tyagi P, Ranjan R (2023). Comparative study of the pharmacological, phytochemical and biotechnological aspects of Tribulus terrestris Linn. and Pedalium murex Linn: an overview. Acta Ecol Sin.

[9] Gulcin İ (2020). Antioxidants and antioxidant methods: an updated overview. Arch Toxicol.

[10] Berkman Z, Tanriover G, Acar G, Sati L, Altug T, Demir R (2009). Changes in the brain cortex of rabbits on a cholesterol-rich diet following supplementation with a herbal extract of Tribulus terrestris. Histol Histopathol.

[11] Gaobotse G, Venkataraman S, Brown PD (2023). The use of African medicinal plants in cancer management. Front Pharmacol.

[12] Patel A, Soni A, Siddiqi NJ, Sharma P (2019). An insight into the anticancer mechanism of Tribulus terrestris extracts on human breast cancer cells. 3 Biotech.

[13] Alshabi AM, Alkahtani SA, Shaikh IA (2022). Tribulus terrestris cytotoxicity against breast cancer MCF-7 and lung cancer A549 cell lines is mediated via activation of apoptosis, caspase-3, DNA degradation, and suppressing Bcl-2 activity. Separations.

[14] Akbaba GB, Öztürkkan FE, Sertcelik M (2021). Evaluation of the cytotoxic effects of ultrasonic extracts of Tribulus terrestris L. on MCF-7 cell line by MTT assay. Hittite j sci eng.

[15] Usman H, Abdulrahman F, Ladan A (2007). Phytochemical and antimicrobial evaluation of Tribulus terrestris L.(Zygophylaceae). Growing in Nigeria. Res J Biol Sci Medwell Journals.

[16] Ptacek G, Shackman J, Ullis K (1999). Super "T": The Complete Guide to Creating an Effective, Safe and Natural Testosterone Enhancement Program for Men and Women. Simon and Schuster.

[17] Vinothini K, Devi MS, Sekar S, George BP, Abrahamse H, van Vuuren BJ, Pandian A (2018). In Vitro Plant Regeneration, Comparative Biochemical and Antioxidant Potential of Calli and Seeds of Sesbania grandiflora (L.) Poiret. Medicinal Plants.

[18] Gacem MA, Telli A, Gacem H, Ould-El-Hadj-Khelil A (2019). Phytochemical screening, antifungal and antioxidant activities of three medicinal plants from Algerian steppe and Sahara (preliminary screening studies). SN Appl Sci.

[19] Sivalingam AM, Pandian A, Rengarajan S, Ramasubbu R (2024). Phytochemical profiling and In-vitro antioxidant activity of In-vivo α-amylase and α-glucosidase inhibitory activities of kidney Costus spicatus extract in diabetic Albino Wistar rats [PREPRINT]. Res Sq.

[20] Vimal S, Taju G, Nambi KSN, Abdul Majeed S, Sarath Babu V, Ravi M, Sahul Hameed AS (2012). Synthesis and characterization of CS/TPP nanoparticles for oral delivery of gene in fish. Aquac.

[21] Cury-Boaventura MF, Pompéia C, Curi R (2004). Comparative toxicity of oleic acid and linoleic acid on Jurkat cells. Clin Nutr.

[22] Baali N, Khecha A, Bensouici A, Speranza G, Hamdouni N (2019). Assessment of antioxidant activity of pure graphene oxide (GO) and ZnO-decorated reduced graphene oxide (rGO) using DPPH radical and H2O2 scavenging assays. C.

[23] Gogoi R, Sarma N, Loying R, Pandey SK, Begum T, Lal M (2021). A comparative analysis of bark and leaf essential oil and their chemical composition, antioxidant, anti-inflammatory, antimicrobial activities and genotoxicity of North East Indian Cinnamomum zeylanicum Blume. Natural Products Journal.

[24] Rengarajan S, Thangavel N, Sivalingam AM, Lakshmanan G, Selvakumari J, Pandian A (2023). Green synthesis and characterization of silver nanoparticles with different solvent extracts of Sesbania grandiflora (L.) Poiret and assessment of their antibacterial and antioxidant potentials. Biomass Convers Biorefin.

[25] Sivalingam AM, Pandian A, Rengarajan S (2023). Extraction, biosynthesis, and characterization of silver nanoparticles for its enhanced applications of antibacterial activity using the Silybum marianum Linn. plant. Biomass Convers Biorefin.

[26] Sivalingam AM, Pandian A, Rengarajan S, Ramasubbu R (2023). Polyphenol-compounds from green synthesis of antimicrobial property of silver nanoparticles using Eichhornia crassipes: characterization and applications. Silicon.

[27] Wolf TG, Cagetti MG, Fisher JM, Seeberger GK, Campus G (2021). Non-communicable diseases and oral health: an overview. froh.

[28] Yuan Z, Du W, He X, Zhang D, He W (2020). Tribulus terrestris ameliorates oxidative stress-induced ARPE-19 cell injury through the PI3K/Akt-Nrf2 signaling pathway. Oxid Med Cell Longev.

[29] Zhao J, Tian XC, Zhang JQ, Li TT, Qiao S, Jiang SL (2023). Tribulus terrestris L. induces cell apoptosis of breast cancer by regulating sphingolipid metabolism signaling pathways. Phytomedicine.

[30] Basaiyye SS, Naoghare PK, Kanojiya S, Bafana A, Arrigo P, Krishnamurthi K, Sivanesan S (2018). Molecular mechanism of apoptosis induction in Jurkat E6-1 cells by Tribulus terrestris alkaloids extract. J Tradit Complement Med.

[31] Sirotkin AV, Kolesárová A (2021). Puncture vine (Tribulus Terrestris L.) in control of health and reproduction. Physiol Res.

[32] Pourali M, Yaghoobi MM, Sormaghi MHS (2017). Cytotoxic, anti-proliferative and apoptotic effects of Tribulus terrestris L. fruit extract on human prostate cancer Lncap and colon cancer HT-29 Cell Lines. Jundishapur J Nat Pharm Prod.

[33] Patel A, Bhatt M, Soni A, Sharma P (2021). Identification of steroidal saponins from Tribulus terrestris and their in silico docking studies. J Cell Biochem.

[34] Abbas MW, Hussain M, Akhtar S, Ismail T, Qamar M, Shafiq Z, Esatbeyoglu T (2022). Bioactive compounds, antioxidant, anti-inflammatory, anti-cancer, and toxicity assessment of Tribulus terrestris-in vitro and in vivo studies. Antioxidants.

[35] Lee HH, Ahn EK, Hong SS, Oh JS (2017). Anti-inflammatory effect of tribulusamide D isolated from Tribulus terrestris in lipopolysaccharide-stimulated RAW264.7 macrophages. Mol Med Rep.

[36] Fabová Z, Tarko A, Harrath AH, Alwasel S, Kotwica J, Sirotkin AV (2022). Tribulus terrestris can suppress the adverse effect of toluene on bovine and equine ovarian granulosa cells. Reprod Domest Anim.

[37] Fernández-Lázaro D, Seco-Calvo J, Pascual-Fernández J, Domínguez-Ortega C, Del Valle Soto M, Mielgo-Ayuso J (2022). 6-week supplementation with Tribulus terrestris L. to trained male CrossFit(®) athletes on muscle, inflammation, and antioxidant biomarkers: a randomized, single-blind, placebo-controlled trial. Int J Environ Res Public Health.

[38] Ariyan F, Farshad A, Rostamzadeh J (2021). Protective effects of Tribulus terrestris and Cinnamomum zeylanicum extracts and trehalose added to diluents on goat epididymal sperm freezability. Cryobiology.

[39] Fatima L, Sultana A (2017). Efficacy of Tribulus terrestris L.(fruits) in menopausal transition symptoms: a randomized placebo controlled study. Adv Integr Med.

[40] Al-Bayati FA, Al-Mola HF (2008). Antibacterial and antifungal activities of different parts of Tribulus terrestris L. growing in Iraq. J Zhejiang Univ Sci B.

[41] Dagenais TRT, Keller NP (2009). Pathogenesis of Aspergillus fumigatus in invasive aspergillosis. Clin Microbiol Rev.

[42] Semerdjieva IB, Zheljazkov VD (2019). Chemical constituents, biological properties, and uses of Tribulus terrestris: a review. Natural Product Communications.

[43] Chauhan S, Sharma D, Goel HC (2018). An in vitro evaluation of Tribulus terrestris L. fruit extract for exploring therapeutic potential against certain gut ailments. Indian J Exp Biol.

